# Uranium XAFS analysis of kidney from rats exposed to uranium

**DOI:** 10.1107/S1600577517001850

**Published:** 2017-02-20

**Authors:** Keisuke Kitahara, Chiya Numako, Yasuko Terada, Kiyohumi Nitta, Yoshiya Shimada, Shino Homma-Takeda

**Affiliations:** a Graduate School of Science, Chiba University, 1-33 Yayoi-cho, Inage-ku, Chiba 263-8522, Japan; b National Institute of Radiological Sciences, National Institutes for Quantum and Radiological Science and Technology, 4-9-1 Anagawa, Inage-ku, Chiba 263-8555, Japan; c Japan Synchrotron Radiation Research Institute, Mikazuki, Hyogo 679-5198, Japan

**Keywords:** uranium, µXAFS, elemental mapping, kidney

## Abstract

The kidney is the critical target of uranium exposure because uranium accumulates in the proximal tubules and causes tubular damage, but the chemical nature of uranium in kidney, such as its chemical status in the toxic target site, is poorly understood. Micro-X-ray absorption fine-structure (µXAFS) analysis was used to examine renal thin sections of rats exposed to uranyl acetate. The U *L*_III_-edge X-ray absorption near-edge structure spectra of bulk renal specimens obtained at various toxicological phases were similar to that of uranyl acetate: their edge position did not shift compared with that of uranyl acetate (17.175 keV) although the peak widths for some kidney specimens were slightly narrowed. µXAFS measurements of spots of concentrated uranium in the micro-regions of the proximal tubules showed that the edge jump slightly shifted to lower energy. The results suggest that most uranium accumulated in kidney was uranium (VI) but a portion might have been biotransformed in rats exposed to uranyl acetate.

## Abstract 2

Characterization of uranium accumulated in the micro-regions of renal tubules of rats exposed to uranyl acetate was examined by micro-X-ray absorption fine-structure analysis and the results showed the possible biotransformation of uranium in this *in vivo* system.

## 1. Introduction   

Uranium is an element in the earth’s crust and is found widely in the natural environment. The levels of uranium in surface water and groundwater are generally low (about 0.003–0.1 ppb; Uchida *et al.*, 2006 ▸), but some areas, such as northern Europe (Kurttio *et al.*, 2002 ▸; Seldén *et al.*, 2009 ▸) and North America (Mao *et al.*, 1995 ▸; Magdo *et al.*, 2007 ▸), contain high levels of uranium in groundwater (several ppb to several ppm). The health effects of chronic ingestion of uranium *via* groundwater cannot be ignored in these areas because the ingestion of uranium at high environmental levels can induce kidney dysfunction (Magdo *et al.*, 2007 ▸). Depleted uranium produced as a by-product in the manufacture of nuclear fuel was used in weapons during the Gulf wars of the 1990s (McDiarmid *et al.*, 2004 ▸). This depleted uranium continues to contaminate these areas and thus the inhabitants are in danger of long-term exposure to uranium. Uranium enters the body and forms the complex uranyl hydrogencarbonate in blood, which diffuses throughout the body and then accumulates in kidney, bone and liver (Moss, 1985 ▸). The kidney is the critical target for uranium toxicity (Leggett, 1989 ▸). Uranium-induced renal toxicity is characterized by renal tubule damage (Leggett, 1989 ▸; Fujigaki *et al.*, 2006 ▸) similar to that caused by hazardous heavy metals such as mercury (Homma-Takeda *et al.*, 1999 ▸) and cadmium (Ishido *et al.*, 1998 ▸). However, details regarding the toxic mechanism, and particularly information about the chemical status of uranium at the toxic target site in kidney, require further investigation.

Uranium minerals contain both tetravalent and hexavalent uranium. The reduction of uranium (VI) to uranium (IV) was studied using mackinawite, a reduced iron (II) mono­sulfide (FeS); under anoxic conditions, mackinawite reacted with dissolved uranium (VI) to form surface complexes of uranium (IV) (Gallegos *et al.*, 2013 ▸). The bioreduction of uranium by a wide variety of bacteria has been documented (Anderson *et al.*, 2003 ▸; O’Loughlin *et al.*, 2003 ▸; Suzuki *et al.*, 2003 ▸; Francis & Dodge, 2008 ▸). Uranium (VI) may be reduced in the body (George *et al.*, 2011 ▸), but uranium (IV) has not been identified in mammalian tissues to date. If uranium (VI) is reduced to uranium (IV), for example, uranium could easily precipitate or form stable ionic species. The resulting change in uranium valence could result in its interaction with biological molecules, especially in the target site in the kidney. Accordingly, understanding the mechanism of uranium toxicity requires identifying the chemical form of uranium in the kidney.

We recently used synchrotron radiation X-ray fluorescence analysis with a microprobe to reveal the site-specific accumulation of uranium in micro-regions of renal tubules after exposure of rats to uranium (Homma-Takeda *et al.*, 2015 ▸). In the present study, we attempted micro-X-ray absorption fine-structure (µXAFS) measurements of rat kidney specimens to characterize the uranium species concentrated in micro-regions of renal tubules, in addition to X-ray absorption fine-structure (XAFS) measurements of bulk rat kidney.

## 2. Materials and methods   

### 2.1. Animals and treatments   

Wistar male adult rats (9 weeks old) were obtained from CLEA Japan (Tokyo, Japan). The animals were acclimated to the controlled temperature (22 ± 2°C), humidity (50 ± 10%) and day/night cycle environment (light 07:00–19:00 h) for 1 week before initiating the study. Uranyl acetate (TAAB Laboratories Equipment Ltd., Aldermaston, UK) was dissolved in saline and administered to the animals by subcutaneous injection at 0.5 mg kg^−1^ body weight at 10 weeks of age. For experiments involving prepubertal animals, Wistar female rats (day 19 of gestation; CLEA Japan) were obtained and litters were culled to six pups (male) per dam at two days after birth. Uranyl acetate was administered to the animals at 25 days (weaning at 22 days). The animals were sacrificed at 1, 3 or 15 days after administration and the kidneys were removed (*n* = 3 per time point per group). The animals were treated according to the ‘Guide for the Care and Use of Laboratory Animals in the National Institute of Radiological Sciences’, Japan.

### 2.2. Sample preparation   

#### 2.2.1. Preparation of renal specimens   

One kidney removed from each rat was fixed in 10% neutral-buffered formalin and embedded in paraffin, then cut into 2 mm-thick slices with a microtome.

The other kidney was divided in half. One half was immediately embedded in optimal cutting temperature compound (OTC) and frozen in liquid nitrogen, then cut into 10 µm- or 50 µm-thick slices with a cryo-microtome and placed on polypropyl­ene film. Serial sections were placed on slides and stained with hematoxylin and eosin (HE) to distinguish the nephron unit using an optical microscope.

A portion of the center area (100 mg) of the other half of the kidney was digested with 0.5 ml of nitric acid at 90°C for 30 min using a microwave oven. The digest was diluted with ultrapure water and used for quantitative analysis of the uranium concentration using inductively coupled plasma-mass spectrometry (ICP-MS) (Model SPQ9700, Seiko Instruments Inc., Japan).

#### 2.2.2. Preparation of uranium standards for XAFS analyses   

Uranium adsorbed on cellulose phosphate resin (Whatman International Ltd, UK) and seven types of metal scavengers on silicate particles (SiliaMetS, SiliCycle Inc., Canada) were prepared as standard materials for XAFS analyses. Each metal scavenger consisted of silicate particles with a surface functional group: silicate particles with ethyl­enedi­amine­tetra­acetic acid (Si-TAAcOH), imidazol (Si-Imidazole), amine (Si-Amine), 2,4,6,-trimercaptotriazine (Si-DMT), cysteine (Si-Cysteine), thio­urea (Si-Thio­urea) and thiol (Si-Thiol). Uranyl acetate was incubated with the functionalized cellulose resin or silicate particles under biological conditions. Briefly, the uranium reaction solution was prepared by adding 0.042 mol L^−1^ uranyl acetate and 0.010 mol L^−1^ NaHCO_3_ to 0.10 mol L^−1^ Tris buffer solution (pH 7.4). The molar amount of uranium in the reaction solution was three times the molar concentration of functional group in 5 mg of each functionalized particle. The functionalized particles were incubated with the uranium reaction solution for 1 min, centrifuged for 5 min at 12000*g*, and then the supernatants were removed. The concentration of bound uranium was determined by ICP-MS. The remaining particles were washed three times with 0.010 mol L^−1^ NaHCO_3_/0.1 mol L^−1^ Tris buffer solution, freeze-dried, and then thinly coated onto a piece of scotch tape for XAFS analysis.

### 2.3. µSR-XRF analysis   

Synchrotron radiation induced micro-X-ray fluorescence (µSR-XRF) measurements of the two-dimensional distribution of uranium in kidney were carried out at BL37XU, SPring-8, Japan Synchrotron Radiation Research Institute, using an energy-dispersive SR-XRF system (Terada *et al.*, 2010 ▸). The X-ray beam was focused onto a 1.2 µm × 1.0 µm beam with a Kirkpatrick–Baez mirror and was monochromated using a Si(111) double-crystal monochromator to 30 keV. Uranium concentration mapping was conducted by moving the sample stage in the *X–Z* direction and each point of the uranium *L*β (renal endogenous rubidium interfered from detection of the uranium *L*α line) fluorescence signal (peak width = 16.1–17.6 keV) was collected with a germanium solid-state detector. The obtained elemental maps were colored from deep blue to red, and classified into 256 from the lower detection limit up to the maximum concentration in linear proportion to the element concentration. The uranium concentration range in the images was calculated using thin uranium standards for microbeam analysis (10 µm; 0–500 µg g^−1^) developed in our laboratory (Homma-Takeda *et al.*, 2009*a* ▸). The standards were analyzed under the same conditions as the samples.

### 2.4. XAFS analysis   

U *L*_III_-edge (17.166 keV) XAFS measurements of the bulk kidney specimens were carried out at BL01B1, SPring-8. The X-ray beam (1.0 mm × 0.2 mm) was monochromated using a Si(111) double-crystal monochromator. The incident beam intensity was detected using an ion chamber and the fluorescence yield of U *L*α was detected with a 19-element germanium solid-state detector. Prior to the U *L*_III_-edge XAFS measurements, we scanned the kidney specimens using the X-ray beam and collected the U *L*α fluorescence signal. XAFS measurements were performed at the point providing the highest signal intensity.

U *L*_III_-edge µXAFS measurements of the kidney thin sections were carried out at BL37XU, SPring-8. Spectra were recorded in fluorescence mode. The measurement conditions were the same as the SR-XRF measurement conditions, except that the incident X-ray energy was 17.120–17.250 keV. The U *L*α distribution map of a kidney thin section specimen was measured using a 17.250 keV X-ray beam. The points showing the highest X-ray intensity were subjected to XAFS measurements. The XAFS spectrum of uranyl acetate powder was measured as a reference sample. XAFS analyses were performed using the XAFS data processing software *REX2000* (Rigaku Co., Japan).

## 3. Results and discussion   

### 3.1. XAFS analysis of comparative samples   

**Figure 1:**
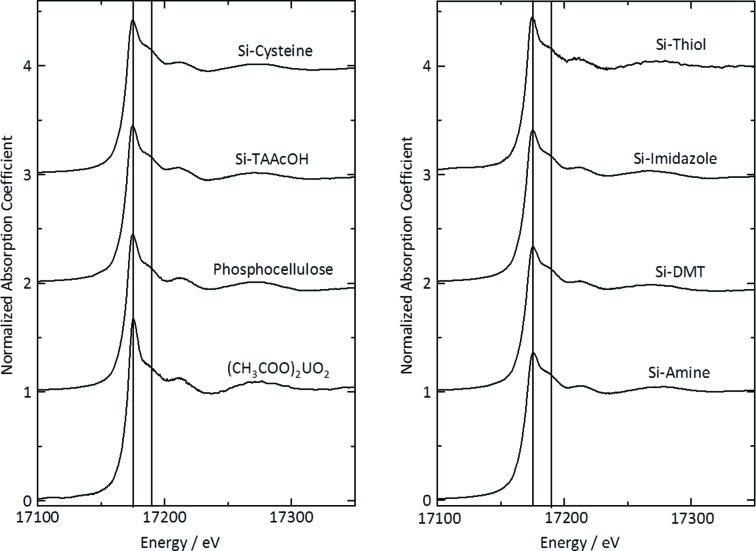
Uranium *L*_III_-edge XANES spectra of the comparative samples. The spectra were normalized to equal intensity at 17250 eV.

**Figure 2:**
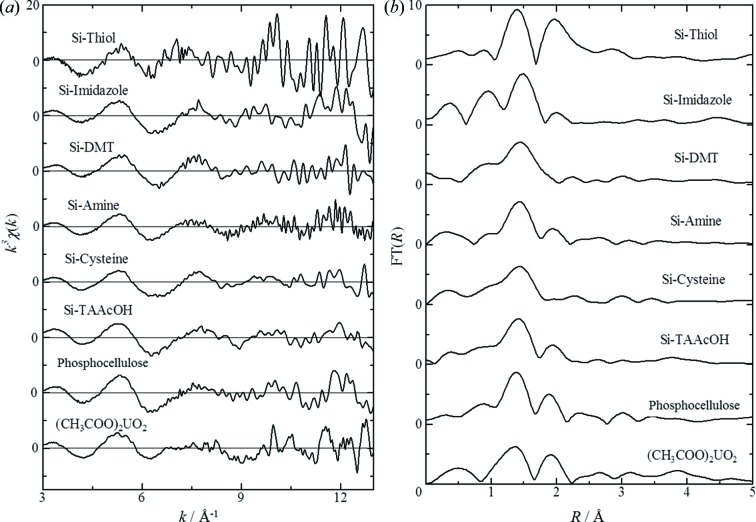
U *L*_III_-edge κ^3^-weighted EXAFS data (*a*) and the corresponding Fourier transforms (*b*).

The concentrations of uranium bound to the functionalized resin or particles were above 15000 µg g^−1^ (Table 1 ▸). X-ray absorption near-edge structure (XANES) spectra of the standard uranium compounds are shown in Fig. 1 ▸. The XANES spectrum of uranyl acetate showed good agreement with a previous report of the XANES spectra of hexavalent uranyl (UO_2_^2+^), with a peak top at 17.175 keV and a shoulder peak at 17.190 keV. These features are due to the short bond between uranium and the axial oxygen atoms in the UO_2_^2+^ unit (U—O_ax_) (Sylwester *et al.*, 2000 ▸). The shapes and peak tops of the XANES spectra of uranium bound to the functionalized resin or particles were similar to those of uranyl acetate. The κ^3^-weighted EXAFS spectra and their corresponding radial distribution functions calculated by Fourier transformation (FT) are shown in Fig. 2 ▸. The κ^3^-weighted EXAFS spectra of all comparative samples showed good agreement with each other in frequency and amplitude of EXAFS oscillations. The oscillation patterns were not complex and might be caused by U—O interaction mainly, and U—U interaction would be very weak. Two major peaks were observed for uranyl acetate, around 1.4 and 1.8 Å, and are believed to arise from the axial oxygen atoms of U—O_ax_ and from oxygen atoms of the carboxyl group in the equatorial plane (U—O_eq_). No information regarding the third coordination was obtained because the X-rays were highly scattered during measurements in fluorescence mode. The peak from U—O_ax_ was observed from all comparative samples. The coordination number was assumed to be U—O_ax_ = 2 and the bond distance was determined to be in the range 1.76–1.82 Å (Table 2 ▸). These results were in agreement with a previous report of the inter-atomic distances in U—O_ax_ (Uehara *et al.*, 2016 ▸), suggesting that hexavalent UO_2_^2+^ bound to the functionalized cellulose resin or silicate particles. The particles with bound uranium might be useful as comparative samples in which the bound UO_2_^2+^ reacts with various biological ligands.

### 3.2. XAFS analysis of bulk rat kidney   

Subcutaneous injection of uranyl acetate to rats (0.5 mg kg^−1^ of body weight) results in renal tubular damage, with the number of proximal tubules containing apoptotic cells increasing on day 2 post-injection, cell deletion from tubules reaching a maximum on day 8, and the appearance of recovery morphology on day 15 (Homma-Takeda *et al.*, 2013 ▸). Higher doses of uranium accelerate renal toxicity and uranium accumulation to kidney (Homma-Takeda *et al.*, 2009*b* ▸, 2013 ▸). In the present study, kidney specimens from rats at the initial phase (day 1 post-injection), the middle phase (onset of toxicity, day 3 post-injection) and the late phase (beginning of recovery, day 15 post-injection) of uranium exposure at high (2 mg kg^−1^ of body weight) and low (0.5 mg kg^−1^ of body weight) doses were subjected to XAFS analysis. The mean renal uranium concentrations (mass of uranium per gram of tissue, µg g^−1^) of these rats are shown in Table 3 ▸.

The optimum conditions and thicknesses of kidney specimens for analysis were identified by measuring XAFS spectra of the paraffin (2 mm) and cryo-sections (10 and 50 µm). The paraffin sections provided indistinct spectra and the absorption edge of the U *L*_III_-edge was indeterminate due to X-rays scattering from the paraffin medium. The U *L*_III_-edge from the 10 µm-thick and 50 µm-thick cryo-sections were clear and thus we used the 10 µm-thick cryo-sections for further XAFS analysis.

**Figure 3:**
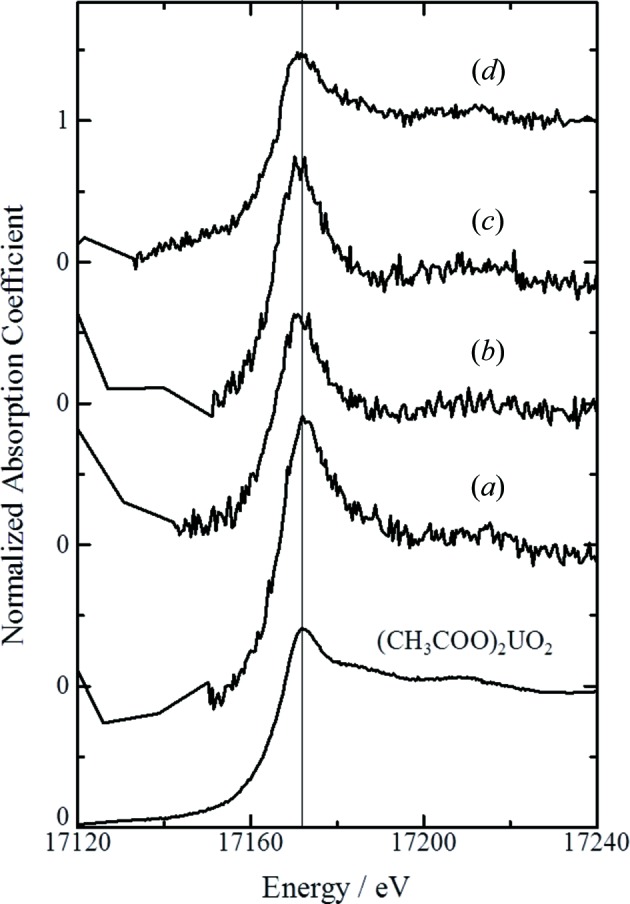
Uranium *L*_III_-edge XANES spectra of rat kidney by bulk kidney measurements. (*a*) Renal section from a prepubartal rat at the middle phase after uranium exposure at the high dose (day 3 post-injection, 2 mg kg^−1^ body weight). (*b*–*d*) Renal section from an adult rat at the initial phase at the low dose [(*b*) day 1 post-injection, 0.5 mg kg^−1^ body weight], the middle phase [(*c*) day 3 post-injection] and the late phase [(*d*) day 15 post-injection] at the high dose (2 mg kg^−1^ body weight). The mean renal uranium concentrations of (*a*), (*b*), (*c*) and (*d*) were 10.2 µg g^−1^, 12.9 µg g^−1^, 34.4 µg g^−1^ and 17.5 µg g^−1^, respectively.

The U *L*_III_-edge XANES spectra of bulk kidney renal specimens obtained at various toxicological phases are shown in Fig. 3 ▸. The mean renal uranium concentrations of these specimens were in the range 10.2–34.4 µg g^−1^ and depended on the dose administered and the toxicological phase at which the kidney was obtained. The U *L*_III_-edge positions for all kidney specimens were the same as the edge position of uranyl acetate, while the peak widths for kidney specimens from the initial and middle phases for adult [Figs. 3(*b*) and 3(*c*) ▸] and prepubertal rats [Fig. 3(*a*) ▸] were slightly narrower. It was previously demonstrated that the edge jump for uranium (IV) shifts slightly to lower energy and the peak widths decrease compared with those of uranium (VI) (Kalkowski *et al.*, 1986 ▸; Uehara *et al.*, 2016 ▸). Our results indicate the possible presence of small amounts of uranium mixed chemical forms, although most of the uranium accumulated in kidney was hexavalent UO_2_^2+^ at all toxicological phases, irrespective of the age of the rat.

Uranium administration to rats results in site-specific accumulation of uranium in the S3 segment of the renal proximal tubules in kidney (Homma-Takeda *et al.*, 2009*b* ▸, 2013 ▸). Highly concentrated uranium, with a maximum uranium concentration 50-fold higher than the mean renal uranium concentration, was observed in micro-regions near the nuclei of cells in the S3 segments (Homma-Takeda *et al.*, 2015 ▸). Microbeam irradiation of the uranium concentrated area would provide clear XAFS spectra without interference from scattered X-rays and thus the concentrated uranium in the S3 segment of the proximal tubules was studied by µXAFS analysis.

### 3.3. µXAFS analysis of concentrated uranium in renal tubules   

**Figure 4:**
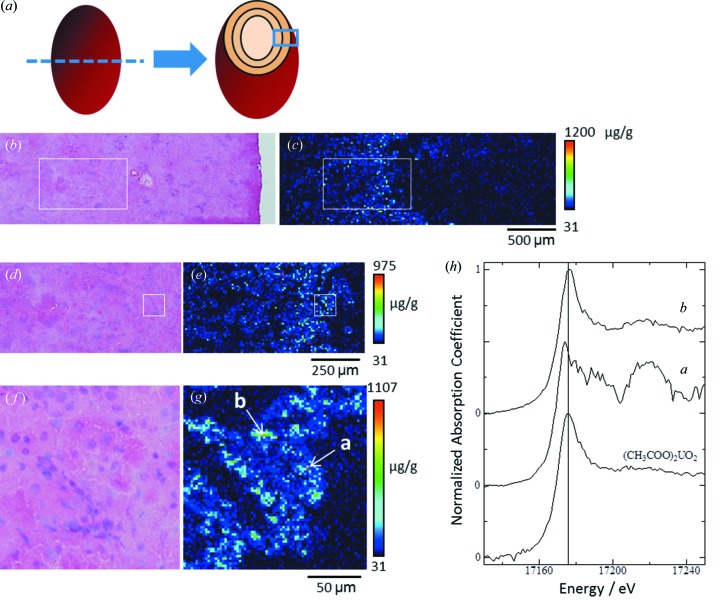
Uranium distribution in kidney and uranium *L*_III_-edge XANES spectra of spots of concentrated uranium in the proximal tubules. The renal section was obtained from an adult rat at the middle phase after uranium exposure at the low dose (day 3 post-injection, 0.5 mg kg^−1^ body weight). (*a*) Diagram of the analyzed area of the renal specimen. (*b*, *d*, *f*) Serial-section stained using hematoxylin and eosin. (*c*) Uranium imaging (150 × 50 steps at 20 µm per step, beam size 1 µm × 1 µm). (*e*) High-resolution uranium imaging of the boxed area in (*b*) and (*c*) (100 × 50 steps at 10 µm per step, beam size 1 µm × 1 µm). (*g*) High-resolution uranium imaging of the boxed area in (*d*) and (*e*) (75 × 75 steps at 2 µm per step, beam size 1 µm × 1 µm). Here, point *a* indicates the first-highest uranium concentrated spot (1114 µg g^−1^) and point *b* indicates the second-highest uranium concentrated spot (948 µg g^−1^) in the analysed area. The periphery of the renal cortex in all images is shown on the right-hand side. The mean renal uranium concentration was 8.46 µg g^−1^. (*h*) Uranium *L*_III_-edge XANES spectra of spots of concentrated uranium; graph *a* is for position *a* and graph *b* is for position *b* in panel (*g*).

Fig. 4 ▸ shows the uranium distribution in an adult rat kidney cross section obtained at the middle phase after exposure to the low uranium dose (0.5 mg kg^−1^ of body weight). The analyzed area shown in Fig. 4(*c*) ▸ corresponds to the diagram in Fig. 4(*a*) ▸ and extends from the outer stripe of the outer medulla to the periphery of the renal cortex. Uranium was detected in the inner cortex and the outer stripe of the outer medulla. The boxed area in Fig. 4(*c*) ▸, in which most of the renal tubules were in the S3 segment of the proximal tubules, was analyzed at high resolution (Fig. 4*e* ▸). Spots of concentrated uranium were scattered in the proximal tubules, with a maximum uranium concentration of around 1000 µg g^−1^ of tissue. The boxed area in Fig. 4(*e*) ▸ was further analyzed at higher resolution [Fig. 4(*g*) ▸] and spots of concentrated uranium were found in the epithelium of the proximal tubules. The first- and second-highest uranium concentrated spots [*a* and *b* in Fig. 4(*g*) ▸], located in the epithelium of the same tubule, were subjected to µXAFS measurements. The uranium levels at positions *a* and *b* were 1114 µg g^−1^ and 948 µg g^−1^, respectively.

The spectral shape and energy position of the peak top for position *b* were almost the same as those for uranyl acetate [Fig. 4(*h*) ▸], whereas the XANES spectrum of position *a* showed a slight negative chemical shift from 17.175 keV to 17.174 keV. It has been reported that reduction of uranium (VI) to (V) or (IV) results in a slight shift of the edge jump at 1–2 eV (Kalkowski *et al.*, 1986 ▸; Uehara *et al.*, 2016 ▸), indicating the possibility of bioreduction of uranium in rats exposed to uranyl acetate. The chemical shift was also observed in other cases, such as adult rats at middle or late phases (data not shown). The induction of oxidative stress after uranium exposure, for example due to the production of reactive oxygen species (Thiébault *et al.*, 2007 ▸) or lipid peroxidation (Linares *et al.*, 2006 ▸), and alterations in the expression of genes related to oxidative stress (Taulan *et al.*, 2004 ▸, 2006 ▸), have been demonstrated in both *in vitro* and *in vivo* systems. The reduction of uranium (VI) in the toxic target site in kidney therefore appears to be a key determinant of uranium renal toxicity. In this regard, studies with not only uranium (VI) but also uranium (V) or (IV) compounds as comparative samples will be required to evaluate the reduction status of uranium in kidney.

## 4. Conclusion   

We analyzed thin sections of kidney from rats exposed to uranyl acetate using µXAFS and observed differences in the chemical status of uranium in spots of concentrated uranium in micro-regions of the proximal tubules. The data suggest that uranium in the proximal tubules might be partially reduced, although the valence of most uranium accumulated in kidney remained unchanged. Understanding uranium renal toxicity requires understanding the bioreduction of uranium in kidney because oxidative stress may mediate uranium toxicity. Therefore, *in situ* µXAFS of renal sections is a useful technique for the study of uranium toxicity.

## Figures and Tables

**Table 1:** Uranium concentrations of the uranium-bound functionalized particles

Sample	Uranium concentration (µg g^−1^)
Cellulose phosphate	15970
Si-TAAcOH	149000
Si-Cysteine	77640
Si-Amine	37400
Si-DMT	87530
Si-Imidazole	74440
Si-Thiol	22500

**Table 2:** EXAFS structural parameters of the comparative samples Errors in distances are ±0.02 Å. Errors in coordination numbers are ±25% and standard deviations as estimated by *EXAFSPAK* are given in brackets.

Sample	Shell	*N*	*R* (Å)	Debye–Wallerfactor (Å^2^)	*R*-factor (%)
(CH_3_COO)_2_UO_2_	O_ax_	2	1.76 (1)	0.06 (1)	2.49
	O_aq_	2.68 (1)	2.38 (4)	0.01 (1)	
Cellulose phosphate	O_ax_	2	1.79 (1)	0.03 (1)	0.29
	O_aq_	3.32 (1)	2.29 (2)	0.07 (0)	
Si-TAAcOH	O_ax_	2	1.80 (1)	0.04 (1)	1.72
	O_aq_	4.63 (4)	2.20 (1)	0.15 (0)	
Si-Cysteine	O_ax_	2	1.79 (1)	0.09 (1)	0.07
	O_aq_	2.08 (1)	2.11 (3)	0.08 (0)	
Si-Amine	O_ax_	2	1.81 (1)	0.04 (1)	0.07
	N	3.34 (2)	2.38 (7)	0.01 (0)	
Si-DMT	O_ax_	2	1.80 (1)	0.07 (2)	0.18
	N	2.12 (2)	2.19 (4)	0.06 (0)	
Si-Imidazole	O_ax_	2	1.82 (1)	0.07 (2)	2.36
	N	2.83 (1)	2.13 (3)	0.06 (0)	
Si-Thiol	O_ax_	2	1.80 (1)	0.02 (3)	0.94
	S	1.96 (1)	2.50 (2)	0.01 (0)	

**Table 3:** Mean renal uranium concentrations after uranyl acetate administration (mean ± standard deviation for three animals)

		Adult			Prepubertal	
Days after administration	Phase of toxicity	Low dose (0.5 mg kg^−1^)	High dose (2 mg kg^−1^)		Low dose (0.5 mg kg^−1^)	High dose (2 mg kg^−1^)
1	Initial	12.2 ± 0.9	42.6 ± 2.3		7.23 ± 1.99	22.7 ± 0.5
3	Middle	8.90 ± 1.14	31.5 ± 2.9		3.47 ± 0.35	9.79 ± 0.44
15	Late	3.02 ± 0.33	18.6 ± 1.6		–	–
